# Effects of different incremental treadmill exercise protocols on the autonomic nervous system in healthy college students: a comparative study based on heart rate variability analysis

**DOI:** 10.3389/fphys.2025.1579929

**Published:** 2025-06-30

**Authors:** Yingying Cao, Shuairan Li, Yi Zha, Xin Yan, Jingdong Jia, Yuanyuan Luo, Aiping Chi

**Affiliations:** ^1^ School of Sports, Xi’an University, Xi’an, China; ^2^ School of Sports, Shaanxi Normal University, Xi’an, China; ^3^ Sports Coaching College, Beijing Sport University, Beijing, China; ^4^ Department of Physical Education, Dazhou College of Traditional Chinese Medicine, Dazhou, China

**Keywords:** incremental load, treadmill exercise regimes, HRV, Poincaré scatter plot, sport intensity

## Abstract

**Objectives:**

This study aimed to examine the acute regulatory effects of three different incremental load treadmill exercise protocols on autonomic nervous system (ANS) function in healthy college students. The ultimate goal was to inform evidence-based training strategies and enhance cardiopulmonary function assessment in this population.

**Methods:**

Forty healthy male college students were recruited to complete three incremental treadmill protocols: Ellestad A, Ellestad B, and Bruce. Participants were equipped with an energy expenditure monitor (GT9-X, ActiLife 1.0), and heart rate (HR) and heart rate variability (HRV) data were recorded at three time points: pre-exercise, immediately post-exercise, and 5 minutes post-exercise. HRV was analyzed using time-domain indices (SDNN, RMSSD, PNN50), frequency-domain indices (LF, HF), and nonlinear metrics derived from Poincaré plots (SD1, SD2), in order to evaluate the impact of exercise intensity on autonomic regulation.

**Results:**

All three protocols resulted in significant reductions in time-domain, frequency-domain, and nonlinear HRV indices compared to pre-exercise baseline values (p < 0.01), indicating marked autonomic suppression. Compared to the immediate post-exercise period, HRV continued to decline following the Ellestad A and B protocols (p < 0.01), while a significant rebound was observed after the Bruce protocol (p < 0.01). Furthermore, the LF/HF ratio progressively increased across the three protocols, revealing a significant main effect of exercise intensity (p < 0.01).

**Conclusion:**

(1) All three incremental treadmill protocols elicited acute HRV alterations, characterized by parasympathetic withdrawal and sympathetic activation, reflecting a transient state of autonomic imbalance; (2) HRV serves as a sensitive physiological marker for detecting exercise-induced fatigue and quantifying training load intensity. Under the Ellestad A and B protocols, heart rate variability (HRV) exhibited a sustained decline throughout the 5-min recovery period, potentially indicating that exercise-induced fatigue load may not have been fully resolved. However, this interpretation warrants further validation through additional objective physiological markers. While the rebound observed after the Bruce protocol may suggest acute autonomic recovery associated with supercompensation and adaptive responses to high-intensity exercise.

## 1 Introduction

Heart rate variability (HRV) is a well-established, non-invasive physiological marker that reflects the dynamic interplay between sympathetic and parasympathetic nervous system activity. It is widely used for monitoring exercise load and fatigue status (Electrophysiology, 1996; Kingsley and Figueroa, 2016). HRV analysis typically encompasses three domains: time-domain metrics (e.g., SDNN, RMSSD, pNN50), frequency-domain metrics (e.g., HF, LF, LF/HF), and nonlinear indices such as SD1 and SD2 derived from Poincaré plots. Among these, RMSSD and pNN50 are closely associated with parasympathetic (vagal) activity, whereas SDNN and the LF/HF ratio reflect overall autonomic modulation (Electrophysiology, 1996; Hsu et al., 2015; Saboul et al., 2013). Previous studies have demonstrated that short-term HRV assessments are effective tools for quantifying training load and identifying fatigue accumulation, particularly in the context of overtraining (Saboul et al., 2013). Prolonged high-intensity training often results in increased sympathetic dominance and reduced vagal modulation, signaling a state of autonomic imbalance (Crawford et al., 2020). Consequently, HRV has gained prominence as a core physiological indicator for prescribing individualized training intensities, evaluating exercise-induced fatigue, and optimizing athletic performance outcomes (Kiss et al., 2016; Plews et al., 2014; Saboul et al., 2013; Uusitalo et al., 2000).

Graded exercise testing (GXT) is a widely adopted approach for evaluating cardiorespiratory fitness and autonomic nervous system (ANS) function through progressively increasing exercise intensity. It plays a pivotal role in developing individualized exercise prescriptions and serves as an objective basis for assessing the effectiveness of sports training and rehabilitation programs (Committee et al., 2012; Demirhan et al., 2014). Among treadmill-based GXT protocols, the Ellestad A, Ellestad B, and Bruce protocols are the most frequently employed. The Bruce protocol represents the highest intensity model and is commonly used to estimate maximal oxygen uptake (VO_2_max). The Ellestad B protocol corresponds to a submaximal load appropriate for the general healthy population, whereas Ellestad A is a moderate-intensity protocol typically applied to individuals with reduced cardiopulmonary capacity. Previous research has shown that all three protocols are associated with reductions in HRV indices, reflecting alterations in autonomic regulation. However, the specific impact of each protocol on ANS function remains a topic of debate, particularly due to the lack of systematic comparative studies focusing on the college student population (Medicine, 1998). As a key target group for university fitness assessments and physical education curricula, the cardiorespiratory capacity and training thresholds of college students are not only vital for evaluating the outcomes of exercise interventions but also essential for ensuring the safety and scientific validity of exercise instruction.

Building on the above rationale, this study recruited 40 healthy male college students to complete three graded treadmill protocols: Ellestad A, Ellestad B, and Bruce. Heart rate variability (HRV) metrics were collected at three time points—pre-exercise, immediately post-exercise, and 5 minutes post-exercise—encompassing indices from the time-domain, frequency-domain, and nonlinear (Poincaré plot) analyses. In addition, the physiological load index (PLI) was used to quantify the intensity of cardiovascular responses. The study aimed to address two primary objectives: (1) To compare the acute effects of the three treadmill protocols on autonomic regulation in college students; and (2) To assess the short-term rebound of HRV following exercise, thereby providing evidence-based insights for fatigue monitoring and identifying optimal recovery windows in the context of university physical education. The findings are expected to contribute novel physiological evidence to support the refinement of collegiate fitness assessment protocols, guide the formulation of individualized exercise prescriptions, and strengthen safety management practices in structured training programs.

## 2 Methods

### 2.1 Participants

Sample size estimation was conducted using G*Power software (version 3.1), with parameters set at an alpha level of 0.05, statistical power of 0.80, and an estimated effect size of f = 0.4 (Brysbaert, 2019). Based on a single-group repeated measures design with three time points, the minimum required sample size was calculated to be 12 participants. To account for potential attrition, an additional 31 individuals were recruited. Following initial screening, three were excluded for not meeting the inclusion criteria, yielding a final sample of 40 healthy male college students with satisfactory physical fitness (see Table 1 for participant characteristics). Participants were recruited through campus-wide public announcements and were screened based on their physical fitness evaluation records obtained during university enrollment. The inclusion criteria were as follows: (1) No history of major organ surgery; (2) Absence of cardiovascular, cerebrovascular, or arrhythmic conditions, with normal electrocardiogram results; (3) No history of musculoskeletal injuries to the limbs or trunk, including muscle strains or serious sports-related injuries; (4) No motor impairments or family history of hereditary diseases; (5) Consistent adherence to a regular lifestyle; (6) No history of sleep deprivation, alcohol use, or medication intake within the week prior to testing, and no emotional disturbances, consumption of strong tea or coffee, or smoking within 24 h before the experiment; (7) Full understanding of the study procedures and associated risks, as confirmed by signed written informed consent. All participants tested negative for drug and alcohol use via urine analysis. This study was approved by the Ethics Committee of Shaanxi Normal University (Approval No. 202316004) and was conducted in strict accordance with the ethical principles outlined in the Declaration of Helsinki for research involving human subjects. Written informed consent was obtained from all participants prior to data collection.

**TABLE 1:** Basic information of experimental participants.

Group	Number(n)	Height (cm)	Weight (kg)	Age (age)	BMI(kg/m^2^)
Boy	40	180.1 ± 5.93	69.52 ± 7.7	20.35 ± 1.31	21.38 ± 1.81

Note: BMI: body mass index.

### 2.2 Study design

This study was designed as a randomized, balanced crossover trial. All participants completed the three graded treadmill exercise protocols in a randomly assigned order. A minimum washout period of 7 days was implemented between each testing session to minimize potential confounding from acute exercise-induced fatigue and carryover effects.

#### 2.2.1 Preliminary testing

One day prior to testing, all participants attended a standardized orientation session that detailed the experimental procedures and outlined key precautions. To minimize potential confounding factors, participants were instructed to maintain adequate sleep and refrain from high-intensity physical activity in the days leading up to the trials. Before the main experimental sessions, participants underwent body composition assessment using a bioelectrical impedance analyzer (InBody 230, Shanghai, China) and completed a maximal oxygen uptake (VO_2_max) test to evaluate their aerobic fitness levels. Following a standardized warm-up, participants performed a cycling test on an Astrand-type aerobic power ergometer (COMBI WELLNESS POWERMAX-VII, Japan), maintaining a pedaling cadence of 50 revolutions per minute. Throughout the test, respiratory gas exchange was continuously monitored using a metabolic analyzer (Parvo Medics, Sandy, UT, United States). The final results confirmed that all participants achieved VO_2_max values exceeding 50 mL/kg/min, indicating high aerobic capacity and confirming their physiological suitability for participation in the graded treadmill protocols.

During the subsequent testing sessions involving the three graded exercise protocols, each participant’s real-time oxygen uptake trajectory (VO_2_ curve) and peak oxygen uptake (VO_2_peak) were continuously monitored to quantify exercise intensity. Exercise intensity was categorized into zones based on the relative intensity metric (%VO_2_max), derived from the measured VO_2_peak. In accordance with the guidelines of the American College of Sports Medicine (Ozemek et al., 2025). Intensity levels were classified as moderate (46%–63%), vigorous (64%–90%), or near-maximal to maximal (≥91%) (Shaffer and Ginsberg, 2017; Tammelin et al., 2002). In addition, the percentage of heart rate reserve (%HRR) was recorded during each session as a secondary index to validate the accuracy of the intensity classification. The age-predicted maximum heart rate (HRmax), calculated using the conventional formula “220 − age,” was used solely for comparative reference to facilitate alignment with previous research findings (Perman et al., 2024).

#### 2.2.2 Experimental equipment and procedures

This study employed a graded exercise testing (GXT) protocol using a high-precision laboratory treadmill system (h/p/cosmos cos10253, Germany). The testing procedures followed three standardized graded exercise protocols, as outlined in Table 2. Protocol 1: Ellestad A. This protocol consisted of four workload stages, each lasting 3 minutes. Treadmill speeds were set at 2.7, 4.8, 6.4, and 8.0 km/h, with a constant incline of 10%. The test began at a speed of 2.7 km/h, with increments of 1.6 km/h per stage, for a total duration of 12 min. Protocol 2: Ellestad B. This protocol extended Ellestad A by adding two additional stages at 8.8 and 9.6 km/h. Starting from the fifth stage, the incline was increased to 15%. Participants were asked to report their Rate of Perceived Exertion (RPE) 30 s after the start of each stage. Protocol 3: Bruce. The Bruce protocol included seven stages, each lasting 3 minutes, with treadmill speeds set at 2.7, 4.0, 5.4, 6.7, 8.0, 8.8, and 9.6 km/h. The corresponding incline levels were 10%, 12%, 14%, 16%, 18%, 20%, and 22%, respectively. RPE scores were recorded every 30 s throughout the test. Participants were instructed to maintain a natural arm swing and to continue the protocol until all stages were completed.

**TABLE 2:** Incremental load running table scheme experiment.

Treadmill exercise	Parameters	Ⅰ	Ⅱ	Ⅲ	Ⅳ	Ⅴ	Ⅵ	Ⅶ
Ellestad A	Running speed (km/h)	2.7	4.8	6.4	8.0	-	-	-
	Gradient (%)	10	10	10	10	-	-	-
	Duration (min)	3	3	3	3	-	-	-
Ellestad B	Running speed (km/h)	2.7	4.8	6.4	8.0	8.8	9.6	-
	Gradient (%)	10	10	10	10	15	15	-
	Duration (min)	3	3	3	3	3	3	-
Bruce	Running speed (km/h)	2.7	4.0	5.4	6.7	8.0	8.8	9.6
	Gradient (%)	10	12	14	16	18	20	22
	Duration (min)	3	3	3	3	3	3	3

Note: Treadmill speed is expressed in kilometers per hour (km/h), and incline is reported as percentage grade (%). Each stage lasted 3 min. “—” indicates that the stage was not included in the corresponding protocol.

**FIGURE 1:**
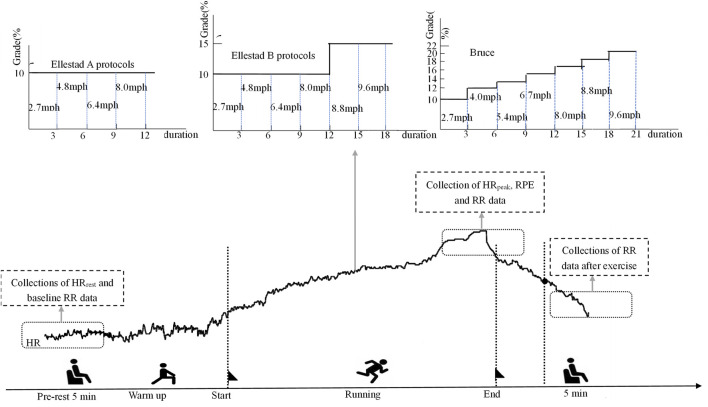
Poincaré scatter plot of lower healthy college students under incremental load model.

All participants successfully completed the three treadmill protocols in sequential sessions (see Figure 1 for the experimental flowchart). To control for circadian variation, all tests were conducted between 9:00 and 11:00 a.m. Participants were instructed to avoid vigorous physical activity for at least 48 h prior to each session. To ensure adequate physiological recovery, only one treadmill protocol was administered per week, with a minimum interval of 7 days between sessions. Throughout both the exercise and recovery phases, participants wore a Polar heart rate monitor and the GT9X system to enable continuous real-time collection of heart rate and heart rate variability (HRV) data. Immediately following each protocol, participants remained seated and rested quietly for 5 minutes, during which HRV data were continuously recorded to assess post-exercise autonomic recovery. All three protocols were administered in strict accordance with standardized procedures under uniform control conditions.

### 2.3 Measurement methods

#### 2.3.1 Methodology for intensity and physiological load analysis

The sequence of the three treadmill protocols was determined using a computer-generated 6 × 6 Latin square design to ensure balanced randomization. A minimum interval of 7 days was maintained between each testing session to minimize potential confounding effects from fatigue or physiological adaptation (Montgomery, 2017). Heart rate (HR) data were collected at three predefined time points—pre-exercise, immediately post-exercise, and 5 minutes post-exercise—using a Polar heart rate monitor in conjunction with the GT9X system. All measurements were conducted in a quiet environment with participants seated in a standardized posture (unsupported back, feet flat on the floor). Key HR metrics included peak heart rate (HR_peak), resting heart rate (HR_rest), and age-predicted maximum heart rate (HR_max), calculated using the formula provided by Gellish et al. (2007). Based on these values, three core physiological indices were computed: (1) percentage of maximum heart rate (HR_max%), (2) percentage of heart rate reserve (HRR%), and (3) Physiological Load Index (PLI). To facilitate comparisons of exercise intensity across treadmill protocols, PLI was employed as a relative indicator of cardiovascular load using the following formula:PLI = (HR_peak + HR_rest)/(2 × HR_rest) This index is not yet standardized and is used in this study solely for internal interpretation of physiological load trends (Tan et al., 2016). In addition to objective physiological indicators, subjective exertion was assessed using the Rate of Perceived Exertion (RPE) scale (Borg, 1982; Swain and Leutholtz, 1997). In addition to objective physiological indicators, subjective exertion was assessed using the Rate of Perceived Exertion (RPE) scale (Borg, 1982; Swain and Leutholtz, 1997). Based on HR_max%, HRR%, PLI, and RPE values, exercise intensity was classified into three levels: Very High Intensity (HRmax%:90%–100%; HRR%:≥ 90; RPE:≥ 18; Heart rate:192–200 192–200 bpm)、High Intensity (HRmax%:76%–90%; HRR% 60%–89%; RPE:14–17; Heart rate:154–190 bpm)、Moderate Intensity (HRmax%:64%–76%; HRR%:40%–59%; RPE:11–13; Heart rate:138–152 bpm) (Ainsworth et al., 2011; Medicine, 2013; Mj, 1957). A PLI value between 1.4 and 1.6 was interpreted as indicative of moderate cardiovascular load. Values exceeding or falling below this range were considered to reflect excessive or insufficient physiological stress, respectively (Liang et al., 2022; Zhao et al., 2022).

#### 2.3.2 Collection and processing of HRV data

R–R interval data were collected using a Polar H10 heart rate monitoring chest strap (sampling rate: 1 kHz; Polar Electro, Finland). Previous research has demonstrated a high degree of concordance between HRV indices derived from this device and those obtained via 3-lead or 12-lead electrocardiograms (ECG), with intraclass correlation coefficients (ICC) ≥ 0.98 under both resting and treadmill exercise conditions (Giles et al., 2016; Gilgen-Ammann et al., 2019). On the day of testing, participants arrived at the laboratory and were fitted with a disinfected Polar H10 strap positioned below the left chest. The device was synchronized to each participant’s profile. Additionally, an Actigraphy GT9X Link device (Beijing Changfeng Technology Co., Ltd.) was placed on the wrist to enable continuous heart rate monitoring throughout the experimental session. During the treadmill exercise protocols, participants’ respiratory frequency naturally increased with exercise intensity until a stable rhythm was achieved. In view of the operational difficulty and potential ventilation–perfusion mismatch associated with paced breathing at moderate to high intensities (Nicolò et al., 2017). Participants were instructed to breathe spontaneously. This ecologically valid approach preserved the authenticity of autonomic responses under real-world exercise conditions. To enhance the reliability of HRV data collected during the resting and recovery phases, respiratory rate was carefully regulated. Participants were instructed to maintain a relaxed breathing pattern—nasal inhalation followed by oral exhalation—at a stable rhythm. External pacing cues were delivered at 0.25 Hz (15 breaths per minute) via visual or auditory aids (e.g., a breathing rhythm light), ensuring consistency while preserving a natural breathing state (Sherlin et al., 2009). Upon completion of each session, heart rate and R–R interval data were exported using ActiLife 1.0 software. The R–R series were then imported into Kubios HRV Premium 3.3.1 (Kubios Oy, Finland) for time-domain, frequency-domain, and nonlinear analysis. In accordance with international guidelines (Electrophysiology, 1996), only low-frequency (LF) and high-frequency (HF) bands were included in the 5-min frequency-domain HRV analysis. To ensure data standardization, stable 5-min segments were extracted from both pre-exercise and post-exercise phases, and the corresponding HRV indices were recorded. A summary of the HRV metrics utilized in this study is presented in Table 3.

### 2.4 Statistical analysis

All statistical analyses were conducted using IBM SPSS Statistics (version 26.0; IBM Corp., Armonk, NY, United States). The Shapiro–Wilk test was first applied to assess the normality of all variables. Variables following a normal distribution were presented as mean ± standard deviation (M ± SD). For non-normally distributed variables, logarithmic transformation was applied to meet the assumptions of parametric analysis (Aguinis et al., 2013). A two-way repeated-measures analysis of variance (ANOVA) with a 3 (time: pre-exercise, immediately post-exercise, 5 min post-exercise) × 3 (intensity: Ellestad A, Ellestad B, Bruce) design was employed to examine both main effects and interaction effects. The order of test protocol administration, based on the six-level Latin square sequence, was included as a fixed between-subjects factor. Studentized residuals exceeding ±3 standard deviations were identified as outliers and excluded from the analysis. Mauchly’s test was used to evaluate the assumption of sphericity for all ANOVA procedures. When this assumption was violated (p < 0.05), the Greenhouse–Geisser correction was applied to adjust the degrees of freedom. In the presence of significant interaction effects, simple main effects analyses were conducted to further examine differences across time points and protocol intensities. Post hoc comparisons were conducted using the Bonferroni correction (Blanca et al., 2023). To additionally control for Type I error inflation due to multiple comparisons, the Bonferroni procedure was applied to all p-values associated with HRV-related main and interaction effects, and corresponding false discovery rate (FDR)-adjusted q-values were calculated. Statistical significance was defined as p < 0.05, with p < 0.01 indicating high statistical significance.

## 3 Results

### 3.1 Basic information of participants

**FIGURE 2:**
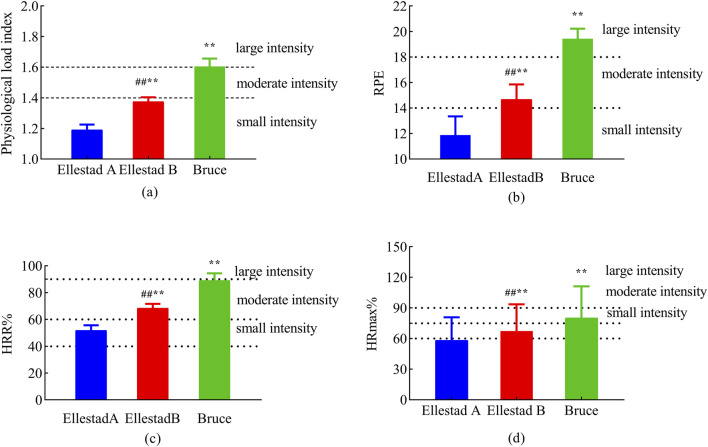
Exercise load assessment of healthy college students under three running platform models. Note: (a: physiological load index; b: RPE; c: HRR%; d: %HRmax) **, p < 0.01 compared with Ellestad B; ##, p < 0.01 compared with Ellestad A.

A total of 43 healthy male college students from Shaanxi Normal University were initially recruited for the study. Following preliminary screening, three individuals were excluded for not meeting the aerobic endurance criteria, resulting in a final sample of 40 participants. As illustrated in Figure 2a, the PLI during the Bruce protocol was maintained within the range of 1.6–1.8. In contrast, the majority of participants in the Ellestad A and B protocols exhibited PLI values below 1.4, indicating comparatively lower physiological load in these two protocols. Significant differences in perceived exertion were observed among the three protocols. The statistical model accounted for the randomized testing order using a balanced Latin square design. Analysis revealed no significant main effect of sequence or interaction with protocol (all p > 0.20), suggesting that the randomization effectively controlled for potential learning effects or residual fatigue bias. Figure 2b presents the RPE scores for each protocol: 11.88 ± 1.47 for Ellestad A, 14.67 ± 1.18 for Ellestad B, and 19.43 ± 0.80 for Bruce. These differences were statistically significant (p < 0.01). As shown in Figure 2c, the corresponding HRR% values were 51.85 ± 3.86 for Ellestad A, 68.40 ± 3.20 for Ellestad B, and 89.13 ± 5.24 for Bruce, also revealing significant inter-protocol differences (p < 0.01). Figure 2d displays the percentage of %HR_max for each protocol, with values of 66.89 ± 2.59 for Ellestad A, 77.44 ± 1.79 for Ellestad B, and 91.99 ± 3.14 for Bruce, again showing significant differences across protocols (p < 0.01). In summary, the Bruce protocol induced significantly greater physiological load and perceived exertion than the Ellestad A and B protocols, underscoring its distinctive role as a high-intensity graded treadmill test.

### 3.2 Heart rate results

**FIGURE 3:**
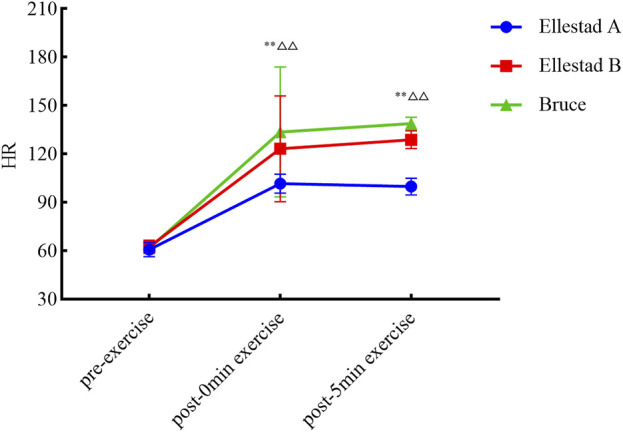
Experimental procedure.

As illustrated in Figure 3, temporal changes in HR across the three graded treadmill protocols were analyzed at different time points. One-way ANOVA revealed no significant differences in resting HR between groups prior to exercise (p > 0.05). However, immediately post-exercise, HR differed significantly across protocols (F (2, 680) = 44.332, p < 0.001). Post hoc multiple comparisons confirmed that all pairwise differences at the 0-min time point were statistically significant (p < 0.01). At 5 min post-exercise, intergroup differences in HR remained significant (F (2, 120) = 429.762, p < 0.001), with subsequent post hoc analyses again indicating significant differences between all protocol pairs (p < 0.01). In summary, the three graded exercise protocols had a significant impact on post-exercise heart rate, with distinct recovery trajectories observed across protocols during the short-term recovery phase.

### 3.3 Time-domain and frequency-domain changes of HRV under different incremental load models

A two-way repeated-measures ANOVA was conducted to examine the effects of time (pre-exercise, immediately post-exercise, and 5 min post-exercise) and exercise intensity (Ellestad A, Ellestad B, Bruce) on time- and frequency-domain HRV parameters. As presented in Table 3, results from the two-way repeated-measures ANOVA revealed significant interaction effects between exercise protocol and time for all time- and frequency-domain HRV indices (p < 0.001), indicating that treadmill exercise intensity significantly modulates the temporal dynamics of autonomic regulation. At baseline, no significant differences were observed among the three protocols across most HRV parameters, except for a moderate effect size in SDNN between Ellestad A and B. Immediately post-exercise, the Bruce protocol elicited the strongest sympathetic activation, as evidenced by significantly elevated low-frequency power (LF) and LF/HF ratio, alongside marked reductions in parasympathetic markers such as RMSSD and pNN50. In contrast, the Ellestad A and B protocols demonstrated only modest sympathetic predominance. At 5 min post-exercise, the Bruce protocol again showed the most pronounced parasympathetic rebound, with significantly higher values of RMSSD, pNN50, and high-frequency power (HF) compared to Ellestad A and B. These changes were accompanied by substantial reductions in LF and the LF/HF ratio, indicating a rapid reestablishment of sympathovagal balance. All between-group differences remained statistically significant after Bonferroni correction, suggesting that while higher-intensity protocols induce acute sympathetic excitation, they may also trigger a stronger parasympathetic response during early recovery. This pattern supports the concept of a “high load–rapid recovery “response in autonomic nervous system regulation.

**TABLE 3:** The outcomes of the impact of various time intervals on HRV time domain indicators under different incremental load model conditions (n = 40).

Metric	Stage	Group 1		Group 2		Cohen’s d	95% CI lower	95% CI upper	p-value	Significant (Bonferroni)	Two-way repeated measures ANOVA (group × test time)		
											F	P	η2
SDNN	Pre-exercise	Ellestad A	3.90 ± 0.20	Ellestad B	4.04 ± 0.32	−0.525	−0.985	−0.065	0.5215	TRUE	97.311	0.000	0.686
		Ellestad A	3.90 ± 0.20	Bruce	3.86 ± 0.46	0.113	−0.34	0.565	0.6154	TRUE			
		Ellestad B	4.04 ± 0.32	Bruce	4.04 ± 0.32	0.454	−0.004	0.912	0.5456	TRUE			
	Post-0 min	Ellestad A	3.31 ± 0.23	Ellestad B	3.15 ± 0.40	0.49	0.031	0.949	0.0313	TRUE			
		Ellestad A	3.31 ± 0.23	Bruce	2.98 ± 0.75	0.595	0.133	1.057	0.0095	TRUE			
		Ellestad B	3.15 ± 0.40	Bruce	2.98 ± 0.75	0.283	−0.172	0.737	0.2097	TRUE			
	Post-5 min	Ellestad A	2.80 ± 0.62	Ellestad B	1.82 ± 0.36	1.933	1.385	2.481	0.0000	TRUE			
		Ellestad A	2.80 ± 0.62	Bruce	4.25 ± 0.60	−2.377	−2.967	−1.786	0.0000	TRUE			
		Ellestad B	1.82 ± 0.36	Bruce	4.25 ± 0.60	−4.911	−5.818	−4.005	0.0000	TRUE			
RMSSD	Pre-exercise	Ellestad A	3.85 ± 0.30	Ellestad B	3.75 ± 0.65	0.198	−0.256	0.651	0.3797	TRUE	66.243	0.000	0.599
		Ellestad A	3.85 ± 0.30	Bruce	3.77 ± 0.54	0.183	−0.27	0.636	0.4152	TRUE			
		Ellestad B	3.75 ± 0.65	Bruce	3.77 ± 0.54	−0.033	−0.486	0.419	0.8814	TRUE			
	Post-0 min	Ellestad A	3.06 ± 0.24	Ellestad B	2.88 ± 0.50	0.459	0.001	0.917	0.0435	TRUE			
		Ellestad A	3.06 ± 0.24	Bruce	2.46 ± 0.95	0.866	0.393	1.339	0.0002	TRUE			
		Ellestad B	2.88 ± 0.50	Bruce	2.46 ± 0.95	0.553	0.092	1.014	0.0155	TRUE			
	Post-5 min	Ellestad A	2.61 ± 0.73	Ellestad B	1.75 ± 0.52	1.357	0.855	1.859	0.0000	TRUE			
		Ellestad A	2.61 ± 0.73	Bruce	4.30 ± 0.71	−2.347	−2.935	−1.759	0.0000	TRUE			
		Ellestad B	1.75 ± 0.52	Bruce	4.30 ± 0.71	−4.098	−4.894	−3.302	0.0000	TRUE			
PNN50	Pre-exercise	Ellestad A	2.96 ± 0.76	Ellestad B	2.93 ± 0.85	0.037	−0.415	0.49	0.8683	TRUE	62.135	0.000	0.583
		Ellestad A	2.96 ± 0.76	Bruce	2.74 ± 1.12	0.23	−0.224	0.684	0.3071	TRUE			
		Ellestad B	2.93 ± 0.85	Bruce	2.74 ± 1.12	0.191	−0.262	0.644	0.3954	TRUE			
	Post-0min	Ellestad A	1.23 ± 0.62	Ellestad B	0.89 ± 1.22	0.351	−0.104	0.807	0.1202	TRUE			
		Ellestad A	1.23 ± 0.62	Bruce	0.21 ± 1.42	0.931	0.455	1.407	0.0001	TRUE			
		Ellestad B	0.89 ± 1.22	Bruce	0.21 ± 1.42	0.514	0.054	0.973	0.0243	TRUE			
	Post-5 min	Ellestad A	0.07 ± 1.46	Ellestad B	0.22 ± 0.55	−0.136	−0.589	0.317	0.5449	TRUE			
		Ellestad A	0.07 ± 1.46	Bruce	3.42 ± 1.17	−2.532	−3.139	−1.925	0.0000	TRUE			
		Ellestad B	0.22 ± 0.55	Bruce	3.42 ± 1.17	−3.5	−4.22	−2.781	0.0000	TRUE			
LF	Pre-exercise	Ellestad A	7.19 ± 0.49	Ellestad B	7.44 ± 0.53	−0.49	−0.949	−0.031	0.6315	TRUE	140.481	0.000	0.760
		Ellestad A	7.19 ± 0.49	Bruce	6.61 ± 0.97	0.755	0.287	1.223	0.5312	TRUE			
		Ellestad B	7.44 ± 0.53	Bruce	6.61 ± 0.97	1.062	0.579	1.545	0.2202	TRUE			
	Post-0 min	Ellestad A	5.82 ± 0.50	Ellestad B	5.12 ± 0.68	1.173	0.683	1.663	0.0000	TRUE			
		Ellestad A	5.82 ± 0.50	Bruce	3.70 ± 1.94	1.497	0.985	2.008	0.0000	TRUE			
		Ellestad B	5.12 ± 0.68	Bruce	3.70 ± 1.94	0.977	0.498	1.455	0.0000	TRUE			
	Post-5 min	Ellestad A	4.80 ± 0.86	Ellestad B	2.70 ± 0.75	2.603	1.988	3.217	0.0000	TRUE			
		Ellestad A	4.80 ± 0.86	Bruce	7.37 ± 1.47	−2.134	−2.701	−1.567	0.0000	TRUE			
		Ellestad B	2.70 ± 0.75	Bruce	7.37 ± 1.47	−4.002	−4.786	−3.218	0.0000	TRUE			
HF	Pre-exercise	Ellestad A	6.59 ± 0.61	Ellestad B	6.67 ± 0.80	−0.112	−0.565	0.34	0.6164	TRUE	68.280	0.000	0.605
		Ellestad A	6.59 ± 0.61	Bruce	6.49 ± 1.15	0.109	−0.344	0.561	0.6284	TRUE			
		Ellestad B	6.67 ± 0.80	Bruce	6.49 ± 1.15	0.182	−0.272	0.635	0.4189	TRUE			
	Post-0 min	Ellestad A	4.70 ± 0.90	Ellestad B	4.24 ± 0.93	0.503	0.043	0.962	0.0274	TRUE			
		Ellestad A	4.70 ± 0.90	Bruce	5.53 ± 1.51	−0.668	−1.132	−0.203	0.0038	TRUE			
		Ellestad B	4.24 ± 0.93	Bruce	5.53 ± 1.51	−1.029	−1.51	−0.547	0.0000	TRUE			
	Post-5 min	Ellestad A	3.71 ± 0.99	Ellestad B	1.53 ± 0.90	2.304	1.721	2.888	0.0000	TRUE			
		Ellestad A	3.71 ± 0.99	Bruce	7.32 ± 1.37	−3.02	−3.682	−2.359	0.0000	TRUE			
		Ellestad B	1.53 ± 0.90	Bruce	7.32 ± 1.37	−4.995	−5.913	−4.077	0.0000	TRUE			
LF/HF	Pre-exercise	Ellestad A	0.58 ± 0.57	Ellestad B	0.72 ± 0.63	−0.233	−0.687	0.221	0.3005	TRUE	5.007	0.000	0.101
		Ellestad A	0.58 ± 0.57	Bruce	0.31 ± 0.90	0.358	−0.097	0.814	0.113	TRUE			
		Ellestad B	0.72 ± 0.63	Bruce	0.31 ± 0.90	0.528	0.068	0.988	0.0208	TRUE			
	Post-0 min	Ellestad A	0.98 ± 0.56	Ellestad B	0.90 ± 0.40	0.164	−0.289	0.617	0.4644	TRUE			
		Ellestad A	0.98 ± 0.56	Bruce	1.83 ± 0.83	−1.201	−1.692	−0.709	0.0000	TRUE			
		Ellestad B	0.90 ± 0.40	Bruce	1.83 ± 0.83	−1.427	−1.934	−0.921	0.0000	TRUE			
	Post-5 min	Ellestad A	1.11 ± 0.87	Ellestad B	1.34 ± 0.60	−0.308	−0.763	0.147	0.1726	TRUE			
		Ellestad A	1.11 ± 0.87	Bruce	0.79 ± 0.64	0.419	−0.038	0.876	0.0647	TRUE			
		Ellestad B	1.34 ± 0.60	Bruce	0.79 ± 0.64	0.887	0.413	1.361	0.0002	TRUE			

Note:A detailed explanation of Table 3 is presented as follows. A two-way repeated-measures ANOVA, was conducted to assess the effects of time (pre-exercise, immediately post-exercise, and 5 min post-exercise) and exercise protocol (Ellestad A, Ellestad B, and Bruce) on HRV, time-domain and frequency-domain indices. All interaction effects were statistically significant (p < 0.001), indicating that the three protocols produced distinct autonomic responses over time. Pairwise comparisons across protocols (see Table 3) yielded the following results: For SDNN, significant differences emerged at multiple time points. At baseline, a moderate effect size was observed between Ellestad A and Ellestad B (Cohen’s d = −0.525, 95% CI [–0.985, −0.065], p = 0.0215), while the comparison between Ellestad B and Bruce approached significance (Cohen’s d = 0.454, p = 0.0456). Immediately post-exercise, SDNN, values in Ellestad A were significantly different from both Ellestad B (Cohen’s d = 0.490, p = 0.0313) and Bruce (Cohen’s d = 0.595, p = 0.0095). Additionally, a large effect size was found between Ellestad B and Bruce (Cohen’s d = 1.053, p < 0.001). All of these differences remained statistically significant following Bonferroni correction (α = 0.049), suggesting that exercise intensity significantly influenced autonomic recovery dynamics. For RMSSD, comparisons immediately post-exercise revealed moderate to large effect sizes. Values in Ellestad A were significantly higher than in Bruce (Cohen’s d = 0.799, 95% CI [0.343, 1.254], p = 0.0012), and Ellestad B also showed higher values than Bruce (Cohen’s d = 0.556, 95% CI [0.099, 1.013], p = 0.0241). However, only the Ellestad A vs. Bruce comparison remained significant after Bonferroni correction. At 5 min post-exercise, RMSSD, values in Bruce were markedly higher than both Ellestad A (Cohen’s d = 1.966, 95% CI [1.416, 2.517], p < 0.001) and Ellestad B (Cohen’s d = 2.789, 95% CI [2.143, 3.435], p < 0.001), with both comparisons surviving correction. These results suggest a stronger parasympathetic rebound effect following the Bruce protocol. For pNN50, significant group differences were evident in both post-exercise stages. Immediately after exercise, Ellestad A showed significantly higher values than Bruce (Cohen’s d = 0.789, 95% CI [0.334, 1.243], p = 0.0013). At 5 min post-exercise, Bruce demonstrated significantly higher values than both Ellestad A (Cohen’s d = 2.500, 95% CI [1.913, 3.087]) and Ellestad B (Cohen’s d = 2.803, 95% CI [2.151, 3.456], p < 0.001), with all comparisons remaining significant after Bonferroni correction. These findings indicate that the Bruce protocol not only induced acute parasympathetic activation but also promoted superior recovery in the delayed phase. For the LF, component, a consistent trend emerged. Immediately post-exercise, values in the Bruce protocol were significantly lower than in Ellestad A (Cohen’s d = 1.379, 95% CI [0.861, 1.897], p < 0.001) and Ellestad B (Cohen’s d = 1.118, p < 0.001). At 5 min post-exercise, Bruce exhibited significantly higher LF, values than Ellestad A (Cohen’s d = 1.984, 95% CI [1.430, 2.537], p < 0.001) and Ellestad B (Cohen’s d = 2.728, 95% CI [2.077, 3.378], p < 0.001). All comparisons remained significant after Bonferroni correction, reflecting a more pronounced sympathetic rebound in the Bruce protocol. Regarding the HF, component, between-group differences were most prominent at 5 min post-exercise. Bruce showed significantly higher HF, values than both Ellestad A (Cohen’s d = 2.897, 95% CI [2.236, 3.559], p < 0.001) and Ellestad B (Cohen’s d = 4.151, 95% CI [3.430, 4.872], p < 0.001), with all differences retaining significance after correction. These findings reinforce the observation that the Bruce protocol elicits robust parasympathetic reactivation during recovery. For the LF/HF, ratio, a significant post-exercise increase was observed, particularly under the Bruce protocol, which showed significantly higher values than Ellestad A (Cohen’s d = 1.701, 95% CI [1.187, 2.215], p < 0.001) and Ellestad B (Cohen’s d = 1.110, p = 0.0021). Both differences remained statistically significant after Bonferroni correction. However, by 5 min post-exercise, the LF/HF, ratio had returned toward baseline, and no significant differences were detected among the groups.

### 3.4 Poincaré scatter diagram

**FIGURE 4:**
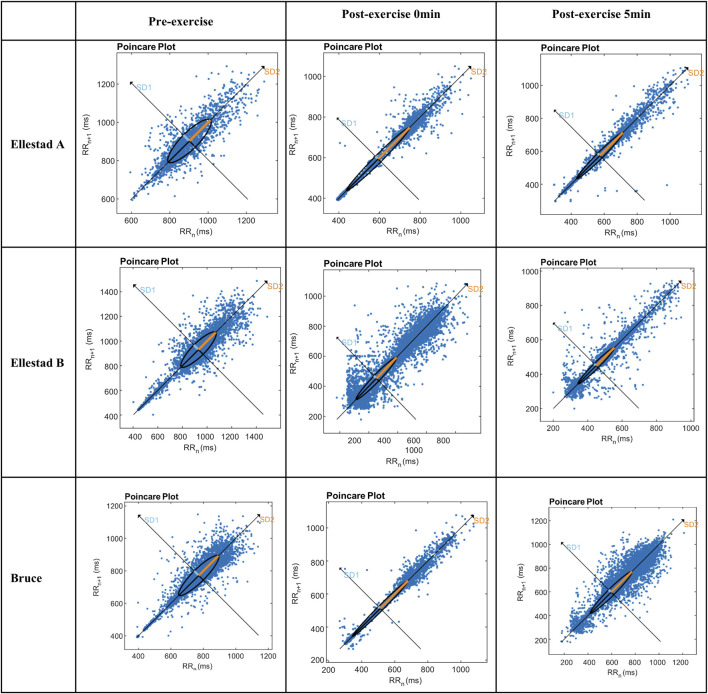
Comparative results of SD1 and SD2 indicators of students under the incremental load model. Note: **, compared with Ellestad A (p < 0.01); △△, compared with Ellestad B (p < 0.01).

The Poincaré plot provides a nonlinear analytical approach for assessing heart rate variability (HRV), in which the major axis (SD2) represents the overall magnitude of variability, while the minor axis (SD1) captures short-term beat-to-beat fluctuations. As shown in Figure 4, the electrocardiographic Poincaré plots of healthy college students at rest display a characteristic comet-shaped distribution, with data points densely aligned along the 45° identity line. As exercise progresses from initiation to the recovery phase, the plot morphology transitions into a narrower, rod-like configuration, reflecting a shift in sympathovagal balance. The experimental results revealed distinct structural changes in the Poincaré plots across different exercise intensities, suggesting that sympathetic activation is strongly influenced by exercise load, leading to functional asymmetry in autonomic control. Despite variations in intensity, all three exercise protocols exhibited a consistent dynamic trajectory: a comet-shaped distribution during rest, followed by a rod-like shape immediately post-exercise and at 5 min post-exercise—indicative of sympathetic dominance and parasympathetic withdrawal. Beyond its visually intuitive depiction of autonomic dynamics, the Poincaré plot also permits quantitative assessment through SD1 and SD2 parameters, further reinforcing its utility in evaluating autonomic regulation under varying physiological conditions.

**FIGURE 5:**
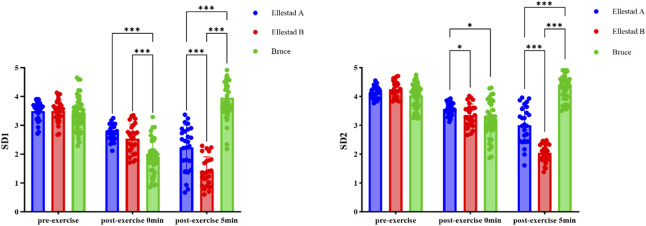
Heart rate result.

As illustrated in Figure 5, both SD1 and SD2 values declined immediately following exercise across all three graded treadmill protocols, with partial a gradual recovery observed at 5 min post-exercise. This trend indicates the onset of autonomic regulatory recovery and a gradual rebalancing of sympathovagal activity. To further investigate the effects of exercise protocol (Bruce, Ellestad A, Ellestad B) and measurement time point (pre-exercise, immediately post-exercise, 5 min post-exercise) on SD1, a two-way ANOVA was conducted. The results revealed a significant interaction effect between protocol and time (F (4, 178) = 96.908, p < 0.001, η^2^ = 0.685). Significant main effects were also observed for time (F (2, 178) = 97.721, p < 0.001, η^2^ = 0.523) and exercise intensity (F (2, 89) = 18.230, p < 0.001, η^2^ = 0.291).

## 4 Discussion

### 4.1 Physiological assessment of different incremental load exercises

Exercise is widely recognized as an effective strategy for preventing cardiovascular disease and enhancing cardiac function. Different exercise intensities place varying demands on energy metabolism and heart rate regulation, making the selection of appropriate intensity crucial for improving cardiopulmonary fitness. Graded exercise testing, which involves the progressive elevation of workload, is commonly used to simulate physiological stress and is widely applied in both fitness assessments and structured training programs. To objectively evaluate the exercise intensity of the three graded treadmill protocols, this study adopted a multi-indicator assessment framework, incorporating the physiological load index (PLI), rating of perceived exertion (RPE), percentage of heart rate reserve (HRR%), and percentage of maximum heart rate (%HRmax). Participants’ physiological responses and exercise tolerance were also taken into account when classifying intensity levels. The results indicated that the Ellestad A protocol corresponds to moderate-intensity exercise, Ellestad B to high-intensity exercise, and the Bruce protocol to maximal-intensity exercise. For healthy college students, the Ellestad A and B protocols imposed manageable workloads and were well-tolerated, whereas the Bruce protocol induced volitional exhaustion within a single session, rendering it more appropriate for maximal cardiopulmonary stress testing.

### 4.2 Immediate heart rate variability (HRV) analysis post progressive load exercise

Heart rate variability (HRV) is closely influenced by an individual’s physical activity status. Extensive research has shown that RMSSD and pNN50 are primary indicators of cardiac vagal modulation, while SDNN reflects overall autonomic regulation by integrating both sympathetic and parasympathetic inputs (Electrophysiology, 1996). In frequency-domain analysis, high-frequency (HF) power is predominantly governed by parasympathetic activity, whereas low-frequency (LF) power and the LF/HF ratio are commonly used to assess sympathovagal balance (Hsu et al., 2015). Additionally, nonlinear analytical techniques, such as Poincaré plot analysis, provide a visual representation of heart rate dynamics and expand the methodological toolkit for evaluating HRV modulation. In this study, HRV responses to exercise were systematically monitored in healthy college students undergoing three graded treadmill protocols: Bruce, Ellestad A, and Ellestad B. The results demonstrated significant reductions in HF, RMSSD, pNN50, and SD1—all key indices of parasympathetic tone. However, participants’ respiratory frequency during the experiment may have influenced the interpretation of parasympathetically mediated HRV indices. Although high-frequency (HF) power was not recalculated in normalized units to control for potential mechanical effects, all HRV assessments were conducted under a paced breathing protocol set at 0.25 Hz,The use of a non-standardized respiratory rate helped ensure consistency across pre- and post-exercise measurements while minimizing variability due to individual breathing patterns. Moreover, several parasympathetic-related HRV indices—including HF, RMSSD, pNN50, and SD1—consistently declined following exercise, collectively supporting the conclusion that the observed vagal withdrawal was not merely a byproduct of respiratory fluctuations. Concurrently, the LF/HF ratio increased significantly, indicating heightened sympathetic activity and diminished vagal modulation. Collectively, these findings suggest that graded exercise disrupts baseline autonomic regulation, resulting in a functional shift toward sympathetic predominance and vagal withdrawal—characteristic markers of acute autonomic imbalance.

Heart rate variability (HRV) is regulated by the dynamic interplay between sympathetic and parasympathetic influences on the sinoatrial node, mediated through both neural and humoral mechanisms. This autonomic coordination is essential for maintaining internal homeostasis and synchronizing heart rate, thereby establishing HRV as a core physiological marker for evaluating cardiopulmonary function (Goldberger et al. 2001; Shaffer et al. 2014). Given its neurophysiological underpinnings, heart rate variability (HRV) is widely recognized as a practical and non-invasive biomarker for assessing cardiopulmonary function. It has been extensively applied in clinical diagnostics, exercise prescription, and rehabilitation outcome evaluation (Huang, 2013). In the present study, physical education students exhibited favorable baseline autonomic function, sufficient heart rate reserve, normal body mass index (BMI), and balanced body composition—demonstrating a solid physiological foundation for tolerating high-intensity exercise testing. These characteristics provided a reliable basis for investigating the effects of exercise protocols of varying intensity. Immediately following exercise, all three graded treadmill protocols induced significant reductions in HRV indices, reflecting a transient autonomic imbalance triggered by acute exercise stress. Among them, the Bruce protocol elicited the most pronounced decline, suggesting that maximal-intensity effort imposes a higher autonomic regulatory load on the cardiovascular system. By 5 minutes post-exercise, most HRV parameters showed partial recovery, indicating the initiation of autonomic rebound and a gradual reestablishment of sympathovagal balance. These findings highlight the dynamic and time-sensitive nature of autonomic regulation following acute physical exertion. Consistent with these findings, Kliszczewicz et al. (2016) reported that high-intensity bodyweight resistance training led to more prolonged vagal withdrawal and heightened sympathetic activation compared to treadmill exercise of matched intensity, suggesting a deeper degree of autonomic disturbance. Similarly, in a randomized crossover study, Gobbo et al. (2024) demonstrated that high-volume, hypertrophy-oriented resistance training significantly increased LF/HF ratios and sympathetic drive during recovery, emphasizing the interaction between training volume and intensity in modulating HRV. Supporting this, a systematic review and meta-analysis by Marasingha-Arachchige et al. (2022) concluded that resistance training at intensities ≥70% of one-repetition maximum (1RM), particularly when paired with high volume and short rest intervals, is more likely to result in acute HRV suppression. Together, these cross-modal findings provide strong physiological and empirical support for the sympathetic dominance–vagal withdrawal response pattern observed under the Bruce protocol in the current study.

The influence of exercise intensity on heart rate variability (HRV) demonstrates a clear dose-dependent pattern. High-intensity exercise has consistently been shown to increase sympathetic markers such as low-frequency (LF) power while concurrently reducing parasympathetic indicators including high-frequency (HF) power, RMSSD, and pNN50 (De Freitas et al., 2015; Lin et al., 2018). As exercise intensity rises, sympathetic activation becomes more pronounced and vagal modulation is further suppressed (Katayama and Saito, 2019). Ma et al. (2019) reported sustained vagal withdrawal and enhanced sympathetic drive, suggesting that cumulative training loads may compromise autonomic regulatory capacity and elevate fatigue risk. The present study’s findings on aerobic exercise support this autonomic response pattern, which appears to generalize across exercise types. While acute high-intensity exercise typically induces sympathetic dominance, its long-term effects may include beneficial autonomic adaptations. For instance, Kingsley and Figueroa (2016) found that 6–12 weeks of periodized resistance training improved resting HRV indices such as RMSSD and HF, underscoring the role of recovery and supercompensation in autonomic remodeling. In line with this, Graessler et al. (2021) demonstrated that endurance, resistance, and high-intensity interval training (HIIT) interventions improved both linear and nonlinear HRV parameters, alongside cardiopulmonary indicators such as VO_2_max, blood pressure, and body composition. These results suggest that while acute sympathetic stress is unavoidable with high-intensity effort, appropriately designed and periodized training can enhance vagal tone and autonomic stability over time. Overall, three key conclusions may be drawn: (1) acute high-intensity exercise generally elicits sympathetic activation and parasympathetic withdrawal; (2) the magnitude of vagal suppression and recovery dynamics depends largely on exercise intensity, volume, and rest interval configuration; and (3) long-term structured training can elevate baseline HRV, improve cardiovascular health, and strengthen autonomic regulation.

In summary, graded exercise at varying intensities exerts distinct regulatory effects on cardiac autonomic function. As exercise intensity increases, elevated cardiac norepinephrine release promotes sympathetic activation and contributes to a global suppression of HRV parameters (Michael et al., 2017). At near-maximal or exhaustive levels, metabolic byproducts—such as intracellular calcium ions (Ca^2+^)—further inhibit vagal tone and reinforce sympathetic dominance. While all three graded treadmill protocols in this study displayed similar trends in immediate heart rate responses and general HRV suppression, deeper analyses revealed protocol-specific differences in autonomic modulation patterns. Accurately identifying the regulatory characteristics associated with different intensity models is essential for developing individualized, precision-based training strategies aimed at optimizing cardiopulmonary adaptability and enhancing the efficacy of exercise interventions. Future research should continue to explore the specific roles of hormones and neurotransmitters in autonomic regulation under varying exercise intensities, thereby advancing our understanding of the neurohumoral mechanisms underlying exercise-induced adaptation. These insights will support the clinical translation and practical application of findings in elite athletic conditioning, public health promotion, and exercise-based rehabilitation.

### 4.3 Analysis of post-exercise heart rate variability (HRV) after 5 min in different incremental load models

In exercise physiology research, the 3–5-min window following physical activity is commonly used to evaluate heart rate variability (HRV) recovery dynamics ( Da Silva et al., 2015; Quigley et al., 2024 ). In the present study, HRV indices exhibited initial recovery within 5 min post-exercise, indicating the onset of autonomic reactivation and rebalancing. This phase was marked by a gradual restoration of parasympathetic tone and a relative withdrawal of sympathetic drive, reflecting a shift from sympathetic dominance toward autonomic equilibrium. Among the three graded treadmill protocols, the Bruce protocol induced the most pronounced HRV suppression, likely due to its steeper workload increments and greater energy and oxygen demands, which intensify myocardial contractility and heart rate, thereby amplifying sympathetic activity (Stanley et al., 2013). However, a notable HRV rebound was observed during early recovery, suggesting that parasympathetic re-engagement plays a critical role in reestablishing cardiovascular homeostasis and autonomic balance. Supporting this, previous studies have shown that both LF and HF components remain suppressed during the first hour following maximal exertion, indicating transient inhibition of both autonomic branches ( Crawford et al., 2020; Katayama and Saito, 2019; Santo, 2005 ). Despite this suppression, many reports describe a rebound phenomenon during recovery, which may represent acute autonomic restoration mechanisms aimed at enhancing energy efficiency and preparing the neuromuscular system for subsequent physical demands (Lin et al., 2016; Plews et al., 2014 ). From the perspective of complex systems theory, exercise serves as an external perturbation that triggers systemic coordination and adaptive transformation, encompassing a dynamic process of stimulus–adaptation–evolution–emergence (Wang et al., 2021). Therefore, the post-exercise increase in HRV not only signifies parasympathetic reactivation but also reflects the body’s capacity for rapid physiological recovery and system-wide reorganization. These dynamic recovery patterns have important implications for post-exercise physiological monitoring and for assessing cardiopulmonary fitness.

Among the various analytical approaches to heart rate variability (HRV), the Poincaré plot serves as a robust nonlinear tool for visualizing cardiac autonomic modulation. In this method, SD1 reflects the parasympathetic system’s capacity for short-term heart rate regulation, while SD2 represents overall heart rate variability, shaped by the combined influences of sympathetic and parasympathetic input (Gifford et al., 2018). In the present study, healthy college students exhibited a classic “comet-shaped” Poincaré distribution at rest—characterized by high long-term variability and low short-term fluctuation—indicative of a balanced autonomic state. As exercise intensity increased and the recovery phase began, the plots transitioned into a compressed, rod-like shape, reflecting impaired autonomic coordination marked by heightened sympathetic activity and reduced vagal modulation. Quantitative analysis confirmed significant declines in both SD1 and SD2 across all exercise intensities, consistent with vagal withdrawal and sympathetic predominance, Toward the end of the 5-min recovery period, both indices began to exhibit signs of partial recovery, suggesting a gradual restoration of autonomic nervous system function. These findings highlight the dynamic interaction between exercise intensity, autonomic regulation, and recovery adaptation, and demonstrate that increasing workload imposes measurable shifts in sympathovagal balance (Buchheit and Gindre, 2006). The behavior of SD1 and SD2 provides valuable insight into stage-specific patterns of HRV response, offering a quantifiable framework to assess exercise-induced autonomic disturbance and subsequent recovery. Overall, the results underscore the practical utility of nonlinear HRV analysis—particularly the Poincaré plot—as an effective method in exercise physiology research, with meaningful applications in individualized training load optimization and post-exercise recovery monitoring. The behavior of SD1 and SD2 provides valuable insight into stage-specific patterns of HRV response, offering a quantifiable framework to assess exercise-induced autonomic disturbance and subsequent recovery.

Overall, the results underscore the practical utility of nonlinear HRV analysis—particularly the Poincaré plot—as an effective method in exercise physiology research, with meaningful applications in individualized training load optimization and post-exercise recovery monitoring.

## 5 Research limitations and future directions

This study has several limitations that should be considered when interpreting the findings and may serve as a basis for methodological refinement in future research. First, although the Polar H10 monitor has been widely validated for HRV data acquisition and is valued for its practicality and ease of use, slight deviations may still occur under certain exercise intensity conditions. To further improve the accuracy and reliability of HRV assessment, future studies may consider employing a simultaneous dual-channel acquisition strategy that integrates the Polar device with standard electrocardiography (ECG) systems. Second, respiratory frequency was neither controlled nor standardized during the experiment, which may have influenced the measurement of HF and RMSSD due to individual variability in spontaneous breathing patterns. This limitation could compromise the interpretive validity of parasympathetically mediated HRV metrics. To improve the precision of HRV analysis, future studies are encouraged to implement paced breathing protocols or concurrently record respiratory frequency and include it as a covariate in the statistical analysis. Moreover, this study did not concurrently assess several commonly used fatigue-related indicators—such as blood lactate concentration, muscular strength decline, and subjective fatigue ratings—which limits the multidimensional characterization of post-exercise fatigue. Future research is encouraged to integrate HRV metrics with a broader range of physiological and perceptual fatigue markers to more comprehensively capture the body’s responses to physical load and fatigue accumulation. Finally, the present study confined its analysis to the first 5 min of post-exercise HRV recovery, which may not fully reflect the complete trajectory of autonomic nervous system restoration. Prior studies have reported that parasympathetic reactivation and delayed sympathetic withdrawal following high-intensity exercise may persist for 30 min or longer (Seiler et al., 2007). Future research should consider extending the post-exercise monitoring window (e.g., to 15–30 min) to allow for a more thorough examination of recovery dynamics under varying exercise intensities, thereby enhancing the applicability of HRV in individualized recovery tracking and training optimization.

## 6 Conclusion

The findings of this study suggest that, within the physiologically tolerable load range for healthy college students, the Ellestad A protocol corresponds to moderate-intensity exercise, Ellestad B to higher-intensity exercise, and the Bruce protocol to maximal-intensity loading. All three graded treadmill protocols significantly impacted autonomic nervous system function, as reflected by enhanced sympathetic activation and reduced parasympathetic modulation. Moreover, higher exercise intensities were associated with prolonged recovery durations required to re-establish sympathovagal balance. Heart rate variability (HRV) indices demonstrated high sensitivity and strong explanatory capacity in response to exercise, effectively capturing variations in training load and exercise-induced fatigue. These findings underscore the physiological relevance of HRV as a robust tool for monitoring autonomic regulation. Notably, the Bruce protocol elicited marked cardiopulmonary stress responses and appears particularly suitable for modeling training adaptations under high-intensity stimuli. Overall, these results provide a scientific basis for individualized cardiopulmonary fitness assessments and support the development of precision-driven training prescriptions.

## Data Availability

The raw data supporting the conclusions of this article will be made available by the authors, without undue reservation.
