# Supplementary material for: Effects of different incremental treadmill exercise protocols on the autonomic nervous system in healthy college students: a comparative study based on heart rate variability analysis
Source: Front Physiol. 2025 Jun 30;16:1579929. doi: 10.3389/fphys.2025.1579929 (PMC12256851; doi:10.3389/fphys.2025.1579929)
Supplement: Supplementary file 1 [file Table1.pdf]

Parameters for HRV Analysis

Index	Significance	Statistical Parameters	Parameter Meaning
SDNN	Standard Deviation of R-R Intervals SDNN	$SDNN = \sqrt{\frac{1}{N}\sum_{i=1}^{N}\left(RR_i - \overline{RR}\right)^2}$	Evaluates the overall variability of HRV throughout the test, disclosing the overall status of the Autonomic Nervous System (ANS)
RMSSD	Root Mean Square of Successive Differences	$RMSSD = \sqrt{\sum_{i=1}^{N}\frac{(\overline{RR}_i - \overline{RR}_{i-1})^2/N}{N-1}}$	Illuminates rapid HRV variations, associated with Parasympathetic Nervous System (PNS) activity; decreased PNS activity correlates with a decrease in RMSSD
PNN50	Proportion of Differences greater than 50ms	$PNN50 = \frac{NN50}{N} \times 100\%$	Indicates PNS activity, decreased PNS activity correlates with a decrease in PNN50
HF	High Frequency	0.15～0.04	Appraises the level of PNS neural activity
LF	Low Frequency	0.04～0.15	Evaluates the regulatory load of PNS and Sympathetic Nervous System (SNS), reflecting SNS activity

LF/HF	Low Frequency/High Frequency	1.5～2.0	Measures the balance between cardiac sympathetic and vagal nerve activity
SD1	Short Axis of Poincaré Scatter Plot; Vertical Coordinate of Standard Deviation of R-R Intervals	$SD1 = \sqrt{\frac{1}{N-1}\sum_{i=1}^{N-1}\frac{(RR_i - RR_{i+1})^2}{2}}$	Denotes the PNS regulation of the heart, determining rapid HR variations.
SD2	Long Axis of Poincaré Scatter Plot; Horizontal Coordinate of Standard Deviation of R-R Intervals	$SD2 = \sqrt{\frac{1}{N-1}\sum_{i=1}^{N-1}\frac{(RR_i + RR_{i+1} - 2\overline{RR})^2}{2}}$	Assessing the combined modulation of SNS and PNS nerves
